# Effects of realistic pesticide mixtures on the springtail *Folsomia candida*

**DOI:** 10.1093/etojnl/vgaf057

**Published:** 2025-03-03

**Authors:** Paula S Tourinho, Zuzana Hochmanová, Petr Kukučka, Olukayode Jegede, Vera Silva, Virginia Aparicio, Jakub Hofman

**Affiliations:** RECETOX, Faculty of Science, Masaryk University, Brno, Czech Republic; RECETOX, Faculty of Science, Masaryk University, Brno, Czech Republic; RECETOX, Faculty of Science, Masaryk University, Brno, Czech Republic; Soil Physics and Land Management Group, Wageningen University & Research, Wageningen, PB, The Netherlands; Soil Physics and Land Management Group, Wageningen University & Research, Wageningen, PB, The Netherlands; INTA, Instituto Nacional de Tecnologías Agropecuaria, Buenos Aires, Argentina; RECETOX, Faculty of Science, Masaryk University, Brno, Czech Republic

**Keywords:** mixture toxicity, plant protection products, soil ecotoxicology, soil invertebrates, collembola

## Abstract

The application of multiple pesticides over the last decades has resulted in their frequent and in some cases long-term presence in soils as complex mixtures. This work assessed the toxicity of realistic pesticide mixtures to the springtail *Folsomia candida* observed in 11 case study sites. Each mixture was composed of five pesticides (as active substances or metabolites), chosen based on their occurrence in soil and expected risk to soil invertebrates. Reproduction tests were conducted in natural agricultural soil, and the springtails were exposed to three concentrations of the selected pesticides: the median environmental concentration (MEC), the predicted environmental concentration (PEC), and five times PEC (5PEC). No significant effect was observed at MEC exposure in any case study sites; however, effects on reproduction, adult survival, and adult size were observed at PEC and 5PEC exposures in five case study sites. Risk quotients (RQs) of individual pesticides were calculated by dividing the exposure concentrations (MEC, PEC, and 5PEC) by the no observed effect concentration values from the literature, and the sum of the five pesticides was calculated as ∑RQ in each case study site. The toxicity at PEC exposure was higher than expected based on the ∑RQ in two case study sites, indicating a possible synergistic mixture effect. This work provides new information on the effects of realistic pesticide mixtures. Further research is required to clarify whether the current risk assessment of individual pesticides adequately protects soil species from exposure to multiple pesticide residues that may occur in even more complex mixtures.

## Introduction

Pesticides are a broad group of chemicals largely used to protect crops against pests. The use of pesticides in agriculture has increased by 50% since the 1990s, reaching a plateau in recent years, with 2.7 million tons of active substances used globally in 2020 (Food and Agriculture Organization of the United Nations [FAO], 2022). In the European Union (EU), strict legislation controls the approval of active substances (a.s.) and authorization of plant protection products (PPP), according to the European Commission (EC; EC, 2009, 2011; European Food Safety Authority [EFSA], 2023). The process is based on a detailed assessment of environmental fate, toxicity, ecotoxicity, and risks for each a.s. at the EU level (EC, 2023a), and then PPP with approved a.s. are accessed at the member state level (EC, 2023b). In Argentina, PPPs are registered in the National Register of Plant Therapeutics, following Food and Agriculture Organization of the United Nations (FAO) specifications for products intended for plant protection (Servicio Nacional de Sanidad y Calidad Agroalimentaria [SENASA], 2024).

Despite existing legislation, phytosanitary products are used in large quantities in both Europe and Argentina and are abused in many situations (Mark et al., 2024). This leads to the widespread occurrence of a.s. and their transformation products in soil, water, sediments, and air compartments (Boye et al., 2019; Chow et al., 2020; Froger et al., 2023; Herrero-Hernández et al., 2020; Kosubová et al., 2020; Silva et al., 2023), biota (Capella et al., 2023; Chandra et al., 2021; Hester et al., 2023; Pelosi et al., 2021; Tongo et al., 2022), and human bodies (Kuang et al., 2020; Mekonen et al., 2023; Souza et al., 2020).

Ecotoxicity testing is an indispensable part of the current EU PPP legislative process. The results of various ecotoxicity tests are required for both a.s. approval (EC, 2013a) and authorization of PPP (EC, 2013b). Soil invertebrates are represented mostly by earthworms and soil macro-/meso-fauna in these requirements. Specific guidelines exist for the testing of soil invertebrates and nontarget arthropods (Candolfi et al., 2001; EC, 2002). Single a.s. are addressed in all these assessments unless the PPP is composed of two or more a.s. and as such tested within the PPP authorization. For PPP applied in tank mixes (i.e., registered and labeled for this use), the risk assessment of the mixture follows the same guidelines as for PPP authorization; however, the sequential or simultaneous serial applications and the environmental mixtures that are created in the soil are not considered in any of these assessments (Frische et al., 2014; Weisner et al., 2021).

The springtail *Folsomia candida* is a standard test species used in risk assessment of pesticides to soil meso- and macrofauna (EC 2013a, 2013b). Moreover, they are one of the most sensitive organisms to pesticide exposure (Joimel et al., 2022; Panico et al., 2022). Single exposure to pesticides may affect springtails at the individual level (i.e., survival, reproduction, and avoidance behavior), but effects on population abundance and species richness have also been reported (Gunstone et al., 2021). When considering pesticide mixtures, most studies focus on binary mixtures (Alves et al., 2014, 2023; Bakker et al., 2022; Pitombeira de Figueirêdo et al., 2019, 2020). Some studies suggest that more than additive effects may occur and that the toxicity on springtail survival and reproduction might not be explained by the sum of individual effects (Alves et al., 2023; Amorim et al., 2012; Santos et al., 2010; Szabó et al., 2023).

Here, we aimed to assess the effects of realistic pesticide mixtures based on residues detected in agricultural soils from 11 case study sites (CSSs) as part of the monitoring program of the SPRINT project (on sustainable plant protection transition; Knuth et al., 2024). Mixtures containing five pesticides were selected in each CSS based on their detection frequency in soil samples (Knuth et al., 2024) and their expected risk to soil invertebrates. Reproduction tests with *F. candida* were performed using the selected mixtures. To better understand the environmental relevance and potential risks of these mixtures, the median environmental concentration (MEC), the predicted environmental concentration (PEC), and five-times PEC (5PEC) exposure as a worst-case scenario were used as exposure concentrations.

## Material and methods

### Selection of pesticide mixtures

The mixture selection used for the ecotoxicity tests was based on the pesticide residues detected in agricultural soils sampled from 11 CSSs as part of a monitoring program of the SPRINT project (Silva et al., 2021). The CSSs were located in 10 European countries and Argentina, including conventional and organic farms (see online supplementary material Table S1). Different crops were selected for each country to cover major crops in Europe (Silva et al., 2021). The soils were sampled during the growing season and 192 pesticides and metabolites were analyzed (Knuth et al., 2024). Full details on soil sampling and findings are published in Knuth et al. (2024).

In each CSS, a mixture of five pesticides was selected based on their frequency of occurrence in the soils and ecotoxicity data to soil invertebrates. The number of pesticides in a mixture was set to five, as several studies have shown that more than 50% of the agricultural topsoils contain from two to 10 residues (Geissen et al., 2021; Hvězdová et al., 2018; Kosubová et al., 2020; Pelosi et al., 2021; Silva et al., 2019). The selection of the pesticides followed a prioritization approach, which is well described in Jegede et al. (2024). Briefly, the pesticides were ranked using their frequency of detection in soils (%; Knuth et al., 2024) and risk quotient (RQ). The RQ was calculated as the ratio between the highest predicted environmental concentrations immediately after application (PEC initial) retrieved from the EFSA reports and the lowest effect concentration (LC/EC50) or the no observed effect concentration (NOEC) in soil invertebrates (e.g., earthworms, springtails, mites, isopods). The reason for considering hazard data for multiple soil invertebrates was that other organisms were also tested for the same mixtures in the SPRINT project (not yet published results). The ecotoxicology data were extracted from the EFSA documents (http://www.efsa.europa.eu), the “Pesticides Proprieties Database” from the University of Hertfordshire (https://sitem.herts.ac.uk/aeru/ppdb/en), and the U.S. Environmental Protection Agency Ecotox database (cfpub.epa.gov/ecotox; see online supplementary material Table S2). The data were adjusted by dividing LC/EC50 and NOEC by factors of 10 and 5, respectively. These “assessment factors” were inspired by the toxicity-exposure ratios from the PPP assessment for soil meso-/macro fauna (EC, 2002) being 10 and five for acute and long-term effects, respectively. The calculated RQ was converted into a percentage relative to the highest RQ observed within each CSS. The pesticides were ranked by multiplying the frequency of detection and the relative risk quotient, and the top five ranked ones were selected for the tested mixture. Pesticides banned before September 2020 were excluded from the selection. The reason is that these substances cannot be officially used during the 2021 season, including the winter crops sown in autumn 2020. Thus, only pesticides that were currently in use in the CSS during the sampling time were considered. In total, 10 fungicides, seven insecticides, seven herbicides, and one metabolite were selected (Table 1; for their properties, see online supplementary material Table S2). The same five pesticides were selected for the CSS in Spain and Italy, so these CSSs were combined for the ecotoxicity tests.

**Table 1.** Selected five pesticides in case study sites (CSSs) from 11 countries, and their concentrations used in the reproduction tests in mg/kg dry soil.

CSS		MEC	PEC	5PEC	CSS		MEC	PEC	5PEC
**SP**	Oxyfluorfen	0.135 ^a^/0.046 ^b^	1.920	9.600	SL	Bixafen	0.010	0.167	0.835
**IT**	Chlorantraniliprole	0.034 ^a^/0.014 ^b^	0.438	2.190		Metolachlor (S)	0.041	1.920	9.600
	Difenoconazole	0.020 ^a^/0.009 ^b^	0.135	0.675		AMPA	0.070	2.036	10.18
	λ-Cyhalothrin	0.025 ^a^/0.006 ^b^	0.033	0.164		Tebuconazole	0.007	0.185	0.925
	Boscalid	0.021 ^a^/0.018 ^b^	0.396	1.980		Terbuthylazine	0.009	1.125	5.625
**PT**	Chlorantraniliprole	0.006	0.438	2.190	CZ	Boscalid	0.018	0.396	1.980
	Boscalid	0.175	0.396	1.980		Thiophanate-methyl	0.160	5.533	27.66
	Glyphosate	0.793	5.760	28.80		λ-Cyhalothrin	0.002	0.033	0.164
	Difenoconazole	0.005	0.135	0.675		Dimoxystrobin	0.017	0.047	0.235
	AMPA	1.910	2.036	10.18		Azoxystrobin	0.014	0.394	1.970
**FR**	Chlorantraniliprole	0.013	0.438	2.190	NL	λ-Cyhalothrin	0.005	0.033	0.164
	Boscalid	0.028	0.396	1.980		Boscalid	0.018	0.396	1.980
	Difenoconazole	0.010	0.135	0.675		Azoxystrobin	0.021	0.394	1.970
	Cyflufenamid	0.004	0.024	0.118		Bixafen	0.022	0.167	0.835
	Glyphosate	0.257	5.760	28.80		Prosulfocarb	0.063	5.333	26.66
**CH**	Difenoconazole	0.009	0.135	0.675	DK	Boscalid	0.008	0.396	1.980
	Methoxyfenozide	0.009	0.091	0.455		AMPA	0.115	2.036	10.18
	Myclobutanil	0.008	0.672	3.360		Diflufenican	0.018	0.250	1.250
	AMPA	0.344	2.036	10.18		Fluopyram	0.008	0.261	1.303
	Pirimicarb	0.023	0.160	0.800		Pendimethalin	0.049	2.133	10.665
**HR**	Boscalid	0.134	0.396	1.980	AR	Glyphosate	0.545	5.760	28.80
	Phosmet	0.006	0.400	2.000		λ-Cyhalothrin	0.006	0.033	0.164
	Acetamiprid	0.010	0.290	1.450		AMPA	1.325	2.036	10.18
	AMPA	0.516	2.036	10.18		Azoxystrobin	0.011	0.394	1.970
	Deltamethrin	0.036	0.022	0.110		Methoxyfenozide	0.012	0.091	0.455

*Note.* SP = Spain; IT = Italy; PT = Portugal; FR = France; CH = Switzerland; HR = Croatia; SL = Slovenia; CZ = Czech Republic; NL = The Netherlands; DK = Denmark; AR = Argentina. MEC = median measured environmental concentration; PEC = predicted environmental concentration; 5PEC = five times PEC)

^a^ and ^b^ represent the MEC values for Spain and Italy, respectively.

### Preparation of the soil contaminated by pesticide mixture

The agricultural soil used in the tests was collected from Unifarm (Wageningen University, The Netherlands). This soil was chosen because it is a natural agricultural soil with low organic content. Organic carbon is an important aspect to consider when testing pesticides, as most agricultural field soils have low organic content, which results in a higher bioavailability of the chemicals (van Gestel, 2012). The soil contained 1% clay (< 2 µm), 8% silt (2–50 µm), and 89% sand (> 50 µm) with a pH_CaCl2_ of approximately 6, organic matter content of 1.3%, total organic carbon of 0.6%, and a maximum water holding capacity (WHC) of approximately 30%. The soil was dried at 70 °C for 24 hr, and stones and other debris were hand-collected.

For each mixture, three concentrations were chosen. The first concentration tested, namely, MEC, was based on the measured concentrations, where each of the five selected substances was added at median measured concentrations in the respective CSS (Knuth et al., 2024). The second concentration was based on the PEC initially taken from EFSA documents (calculations done using a soil depth layer of 5 cm and soil bulk density of 1.5 g/cm^3^). The third tested concentration was five times the PEC value. This was used as the worst-case scenario considering that there might be spots within fields where the initial concentration is higher than modeled (e.g., turn-/end-rows may receive more inputs than the rest of the field). The concentrations, in mg a.s./kg_dry weight_ soil, used in each CSS and treatment are shown in Table 1.

Chemical standards were purchased from Sigma-Aldrich, LGC Standards, and HPC Standards GmbH companies. Stock solutions were prepared in acetone except for glyphosate and aminomethylphosphonic acid (AMPA), which were prepared in distilled water. A scheme of the spiking procedure can be found in online supplementary material Figure S1. One day before the experiment, 8% of the soil (dry wt) was spiked with the pesticides in acetone and left to evaporate overnight. The same amount of solvent was used for all concentrations in a ratio of 8 g soil to 1 ml acetone. The next day, the remaining soil (92% dry wt) was moistened to achieve 50% of the maximum WHC of the total amount of soil (100% dry wt). The amount of glyphosate or AMPA needed to obtain the final soil concentrations was added at this step while moistening the soil to 50% WHC. Subsequently, acetone- and water-spiked soils were mixed thoroughly by hand. Negative control was prepared by adding distilled water (50% WHC), and solvent control was prepared by adding acetone (8 g soil to 1 ml acetone) and distilled water (50% WHC).

### Reproduction test

The springtail *Folsomia candida* was cultured in transparent plastic boxes with a moist bottom of plaster of Paris and charcoal at 20 °C. The organisms were fed with dry yeast (*Saccharomyces cerevisiae*) once a week. Reproduction tests were performed following the Organisation for Economic Co-operation and Development (OECD) Guideline 232 (OECD, 2009). The tests were conducted in glass jars containing approximately 25 g moist soil. Five replicates per concentration were prepared, plus one extra replicate was prepared (no animal added) to be used in the chemical analysis (see online supplementary material Figure S1). At the start of the test, 10 springtails (10–12 days old) were transferred into each jar, and dry yeast was added as food. Test jars were incubated in a climate room at 20 °C and a 16:8-hr light:dark photoperiod. Water loss was replenished weekly, and food was added after 2 weeks. After 4 weeks, the test was ended by adding tap water into the test jars. The content was transferred into a plastic container and stirred carefully to let all the springtails float on the surface. A piece of millimetric paper was placed on the surface and photographs were taken with a digital camera. Adult and juvenile springtails were counted, and the size of the surviving adults was measured using ImageJ software (Ver. 1.53k).

### Determination of pesticides in soils

The initial soil concentration was measured in soil samples frozen (−20 °C) immediately after spiking. The final (i.e., after 28 days) soil concentration was measured in the extra replicate (no animals added) and the soil was frozen at the end of the experiment. The samples were freeze-dried and extracted by the QuEChERS (quick, easy, cheap, effective, rugged and safe) method as described by Silva et al. (2019), and for glyphosate and AMPA extraction, the KOH method was used as described by Yang et al. (2015), Goscinny et al. (2012), and Kaczyński & Łozowicka (2015). For the QuEChERS extraction, 5–10 g dry soil was spiked with D9-Tebuconazole in a 50 ml falcon tube and shaken for 15 min with 15 ml of a mixture of acetonitrile-water (2:1). QuEChERS salts (1.0 g sodium chloride, 4.0 g magnesium sulfate, 1.0 g sodium citrate, 0.5 g sodium hydrogen citrate sesquihydrate) was added into the tubes and manually shaken for 1 min. The tubes were placed in ice for a few minutes until chilled. Then, the samples were centrifuged (5 min, 3,500 rpm, −5 °C) and the supernatants were collected. A volume of 0.5 ml supernatant was diluted in water (1:1) and analyzed by liquid chromatography–tandem mass spectrometry (LC-MS/MS; Agilent 1200 chromatographic system coupled to an ESI/QqQ Agilent Triple Quad 6410 mass spectrometer (Agilent, Santa Clara, CA, USA). Another portion of 0.1 ml supernatant was spiked with PCB 162 and analyzed by gas chromatography (GC) atmospheric pressure ionization mass spectrometry (XEVO TQ-S MS [Waters] coupled to a 7890 GC from Agilent Technologies). The limit of quantification ranged from 0.004–0.05 mg/kg_dry weight_ soil for LC-MS/MS, and from 0.001–0.01 mg/kg_dry weight_ soil for GC-MS/MS.

For analysis of glyphosate and AMPA, 2 g dry soil samples and 10 ml potassium hydroxide (0.6 M) were shaken for 50 min in 50 ml falcon tubes. The samples were centrifuged (30 min, 3,500 rpm, 23 °C), and 0.5 ml supernatant was transferred to a 2 ml plastic vial. In each vial, 40 µL hydrochloric acid was added and 10 µL isotope-labelled standard glyphosate and AMPA solution, followed by 0.25 ml borate and 0.25 ml fluorenylmethoxycarbonyl protecting group and left for 30 min at room temperature. Then 25 µL formic acid was added and the samples were transferred to plastic LC vials integrated with 0.45-μm polytetrafluoroethylene filters. The extracts were analyzed by LC-MS/MS (Agilent, Santa Clara, CA, USA). The limit of quantification was 0.05 mg/kg_dry weight_ soil.

### Data analysis

Adult survival, number of juveniles, and adult size were analyzed by a Kruskal-Wallis one-way analysis of variance test with Dunn’s test, using the FSA package (Ogle et al., 2023) in R studio. For better visualization of the data and to allow the comparison of the effects among the CSS, the data are presented in the graphs as percentages of the respective solvent control treatments.

An RQ for single pesticides was calculated as the ratio between their nominal exposure concentration in the mixture (MEC, PEC, or 5PEC) and NOEC. The nominal concentrations were used because low recoveries from the measured concentrations could overestimate the RQ. The NOEC values for effects on springtail reproduction were retrieved from EFSA reports (conclusions, draft assessment report, and renewal assessment report; see online supplementary material Table S3). For pesticides with octanol-water partition coefficient (Log Pow) > 2, the corrected values given by EFSA were used (i.e., toxicity data corrected by a factor of 2 or no correction if the artificial soil contained 5% organic matter). In some cases, the NOEC values provided by EFSA were only corrected in the toxicity-exposure ratio calculations (e.g., difenoconazole [EFSA, 2011], chlorantraniliprole [EFSA, 2013]), so a factor of 2 was applied for those given NOECs (see online supplementary material Table S3). Values for active substances were used when available, otherwise, data on formulations were used. Prosulfocarb had no available NOEC value, because a test with springtails was not required for the approval of this pesticide, and no value was found in the literature. The RQ for the mixtures in each CSS was calculated by summing up the RQ of the five individual pesticides (∑RQ; see online supplementary material Table S4).

## Results

### Pesticide concentrations in soils

The initial and final soil concentrations can be found in online supplementary material Table S5. Glyphosate and AMPA were detected in control soils with concentrations ranging from 0.05–0.09 and 0.09–0.19 mg a.s./kg_dry weight_ soil, respectively. At MEC, PEC, and 5PEC, the initial measured concentrations of the pesticides ranged from 0%–159%, 1.7%–143%, and 29%–163% of the nominal concentrations, respectively (see online supplementary material Table S5), except for deltamethrin and lambda-cyhalothrin where measured concentrations were 234%–433% higher than nominal concentrations. Aminomethylphosphonic acid also had high recoveries in Slovenia, where the nominal MEC of 0.07 mg a.s./kg_dry weight_ soil was lower than the background concentrations in soil. Most samples (75%) showed recovery below 80% of nominal concentrations, 31% of samples had recoveries ranging from 80%–120%, and 13% showed recoveries above 120%.

The degradation throughout the test was rather low for most of the pesticides, and final concentrations were ≥ 80% of their initial measured concentrations (see online supplementary material Figure S2). These pesticides have half-life (DT50) higher than the test duration of 28 days, and therefore low degradation was expected (see online supplementary material Figure S2). The pesticides with lower DT50 (0.5 to 6.7 days), namely, thiophanate-methyl, acetamiprid, phosmet, and prosulfocarb, showed substantial degradation, with final concentrations < 30% of the initial concentration. The exception was lambda-cyhalothrin, for which the final concentrations were on average only 17% of the initial concentration in contrast with its high DT50 of 174 days.

### Toxicity tests

The toxicity test met all validity criteria according to the OECD guidelines. In control treatments, adult survival was > 80%, the number of juveniles per test jar was > 100, and the coefficient of variance for the juvenile number was < 30%.

**Figure 1.**
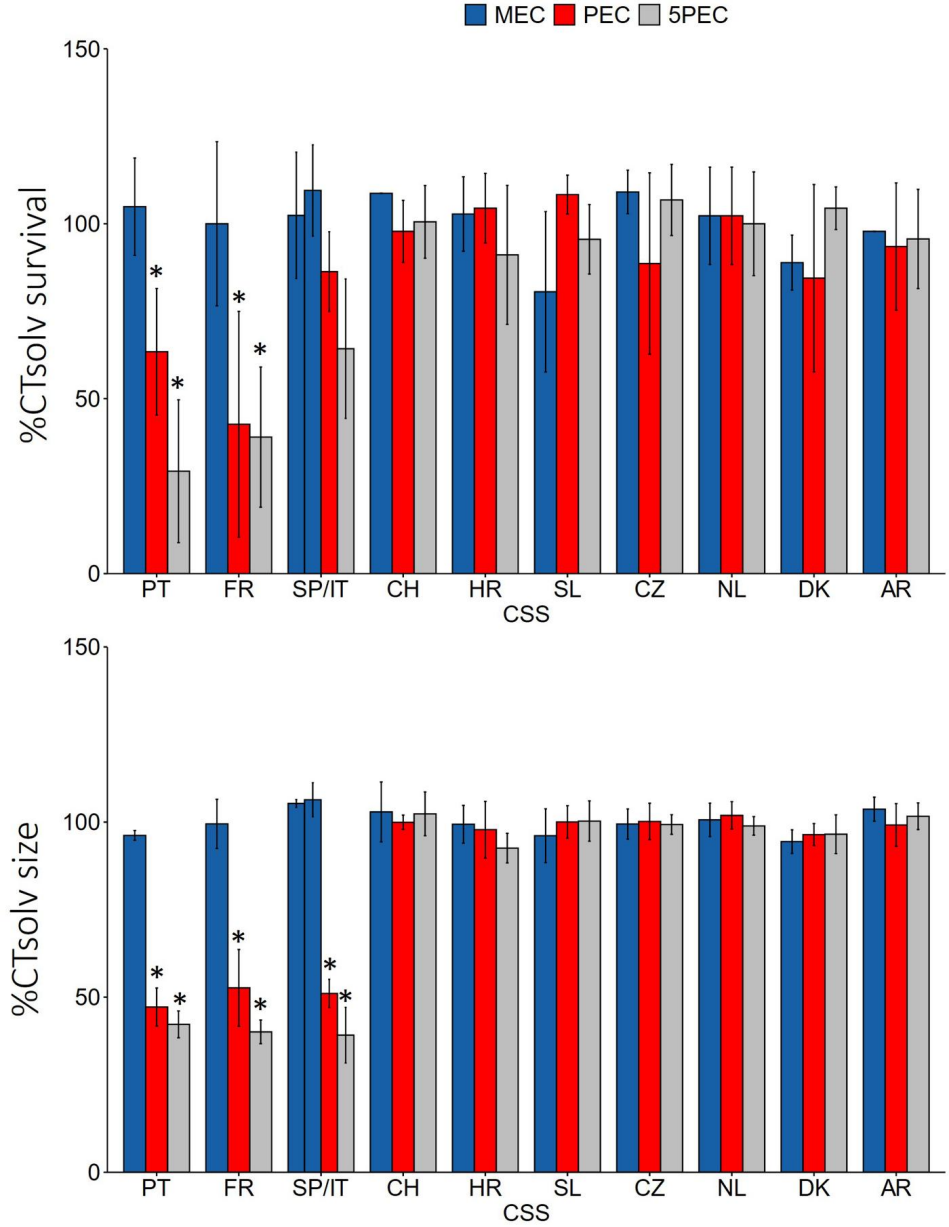
Effects (expressed as percentage of solvent control [CT solv]) of pesticide mixtures on springtail survival (top) and adult size (bottom) in crop systems from 11 countries exposed to median environmental concentration (MEC), predicted environmental concentration (PEC), and five times PEC (5PEC) for 4 weeks. Asterisks indicate a significant difference from the solvent control (Dunn’s test, *p* < 0.05). SP/IT = Spain and Italy (MEC column on the left for SP, MEC column on the right for IT); PT = Portugal; FR = France; CH = Switzerland; HR = Croatia; SL = Slovenia; CZ = Czech Republic; NL = The Netherlands; DK = Denmark; AR = Argentina.

No significant difference was observed between negative and solvent controls for any endpoint (Dunn’s test, *p* > 0.05). Solvent control variation from negative control was 85%–102% for survival, 96%–102% for size, and 81%–98% for reproduction. For this reason, we compared the treatments with the solvent control only. The effects of mixtures on adult survival and size can be found in Figure 1 (for complete raw data, see online supplementary material Figure S2). Springtail survival significantly decreased at PEC and 5PEC treatments in mixtures from Portugal and France when compared with their respective controls (Dunn’s test, *p* < 0.05). The size of surviving adults decreased significantly at PEC and 5PEC in mixtures from Spain/Italy, Portugal, and France (Dunnett’s test, *p* < 0.05).

**Figure 2.**
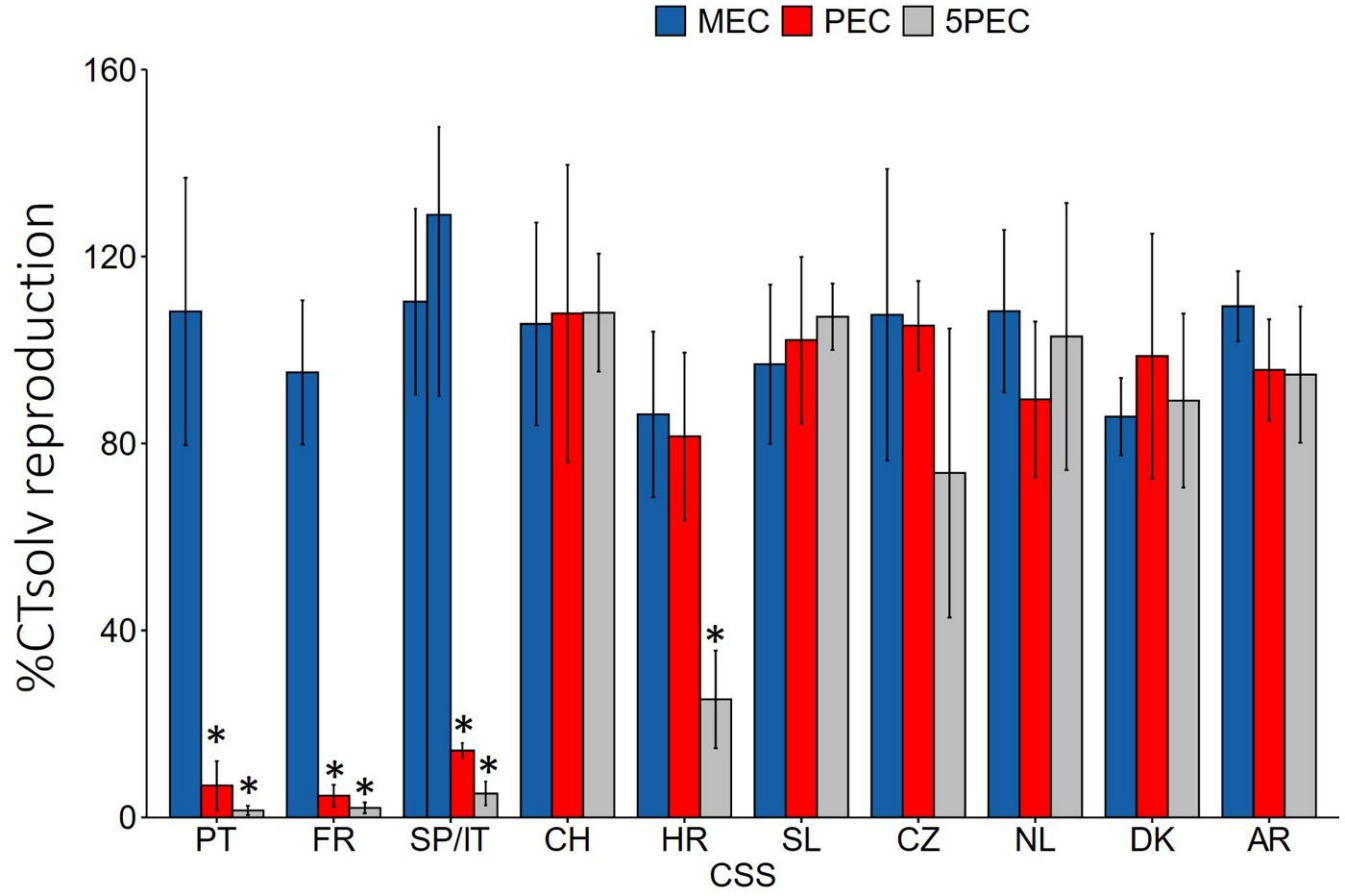
Effects (expressed as percentage of solvent control [CT solv]) of pesticide mixtures on springtail reproduction in crop systems from 11 countries exposed to median environmental concentration (MEC), predicted environmental concentration (PEC), and five times PEC (5PEC) for 4 weeks. Asterisks indicate a significant difference from the solvent control (Dunn’s test, *p* < 0.05). SP/IT = Spain and Italy (MEC column on the left for SP, MEC column on the right for IT); PT = Portugal; FR = France; CH = Switzerland; HR = Croatia; SL = Slovenia; CZ = Czech Republic; NL = The Netherlands; DK = Denmark; AR = Argentina.

The effects of pesticide mixtures on springtail reproduction can be found in Figure 2 (for complete raw data, see online supplementary material Figure S2). The reproduction significantly decreased in animals exposed to PEC and 5PEC treatment in the mixtures from Spain/Italy, Portugal, and France, and 5PEC treatment in the mixture of Croatia (Dunnett’s test, *p* < 0.05).

**Figure 3.**
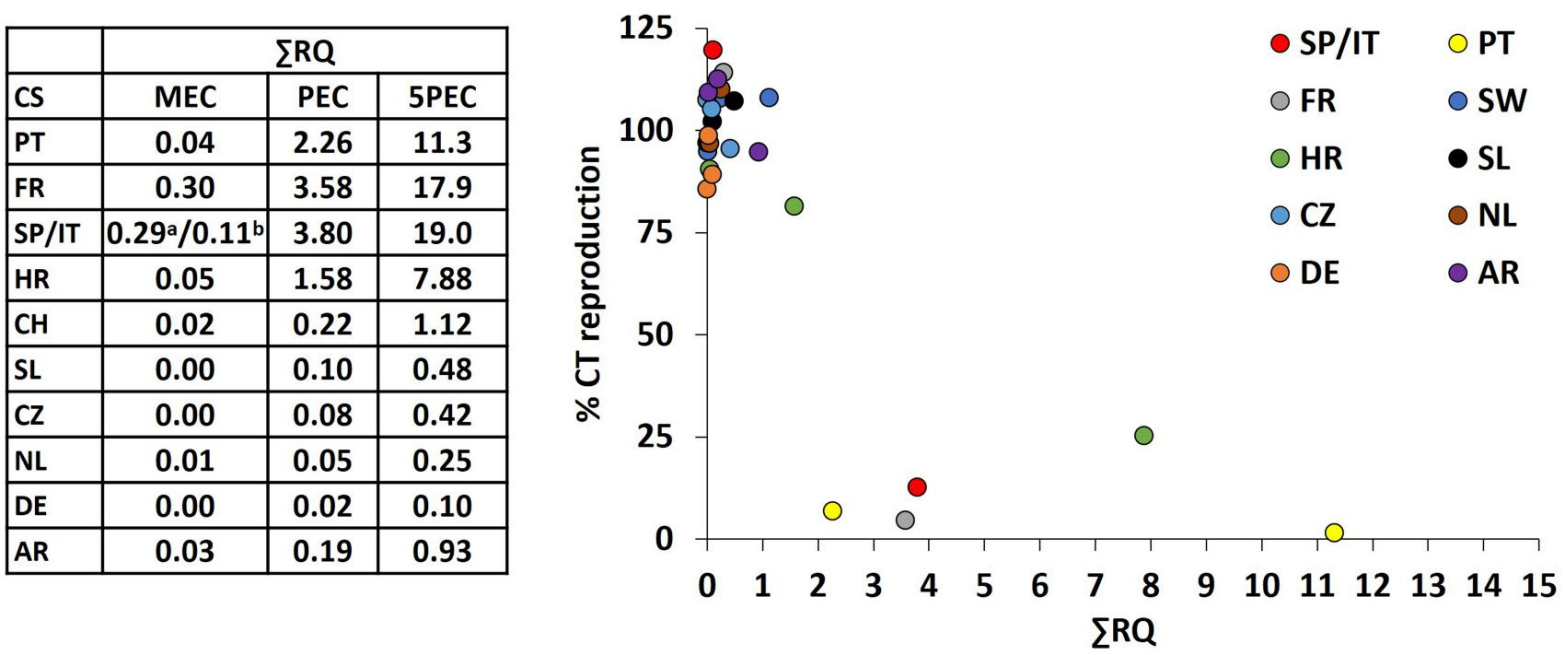
Sum of risk quotient (∑RQ) in the case study sites (CSSs) calculated as the ratio between exposure concentration and NOEC values. The median environmental concentrations (MEC) predicted environmental concentration (PEC), and five times PEC (5PEC). Plot of effects on reproduction (expressed as percentage of solvent control) versus the ∑RQ. ^a^ and ^b^ = the MEC values for Spain and Italy, respectively; SP = Spain; IT = Italy; PT = Portugal; FR = France; CH = Switzerland; HR = Croatia; SL = Slovenia; CZ = Czech Republic; NL = The Netherlands; DK = Denmark; AR = Argentina.

The ∑RQ, based on NOEC values for reproduction, was calculated for each exposure concentration, and the effects on reproduction (expressed as percentage control) were plotted against ∑RQ (Figure 3). Values of ∑RQ were more than 1 for PEC and 5PEC in Spain/Italy, Portugal, France, and Croatia and for 5PEC in Croatia. In these treatments, ∑RQ ranged from 1.12 to 19.0.

## Discussion

### Pesticides in soil

This work investigated the effects of pesticide mixtures from different CSS on a nontarget species, the springtail *F. candida*. The initial and final concentrations of the pesticides were quantified in soil samples after spiking and at the end of the experiment. Relatively low recovery (< 80% of nominal concentrations) was observed in most of the samples. It is difficult to pinpoint the reasons for the low recovery. This may be due to matrix effects or extraction efficiency of pesticides that present different properties (Bakanov et al., 2023).

#### Toxicity results

In six out of 11 CSS, no toxicity on adult survival, size, and reproduction was observed, namely, those from Switzerland, Slovenia, Denmark, Argentina, the Czech Republic, and The Netherlands. These outcomes were expected because the NOEC values of single pesticides were higher than their highest exposure concentrations (5PEC) and the ∑RQ was ≤ 1 (Figure 3).

In the other five CSS, toxic effects were observed on springtail survival, size, and/or reproduction. Reproduction was the most sensitive endpoint, with a significant decrease observed in five CSS (Spain/Italy, Portugal, France, and Croatia). Adult survival and size were significantly affected in four CSS (Spain/Italy, Portugal, and France).

Significant effects on reproduction were observed at PEC and 5PEC in Spain/Italy, Portugal, and France and 5PEC in Croatia. In these treatments, the ∑RQ values were all more than 1 and therefore these results were somewhat expected. The ∑RQ ranged from 2.26 to 19.0 in these CSS, which was a result of one or two pesticides in the mixture with high RQ values (see online supplementary material Table S4) and for which the exposure concentrations exceeded the NOEC values. All mixtures that had significant effects on reproduction had at least one pesticide with RQ more than 1. This indicates that the toxicity was driven mainly by one or two pesticides in each mixture. The most toxic mixtures, Spain/Italy, Portugal, and France, all had in common the insecticide chlorantraniliprole. The PEC of chlorantraniliprole (0.43 mg a.s./kg_dry weight_ soil) was more than two times higher than its NOEC of 0.19 mg a.s./kg_dry weight_ soil. Another example was the herbicide oxyfluorfen, present in Spain/Italy mixtures, which had PEC and NOEC of 1.95 and 1.25 mg a.s./kg_dry weight_ soil, respectively. In Croatia, the 5PEC values of insecticides phosmet and acetamiprid were 2.0 and 1.45 mg a.s./kg_dry weight_ soil, whereas NOEC values were 0.81 and 0.27 mg a.s./kg_dry weight_ soil, respectively, explaining the effects on reproduction observed at this concentration.

It is difficult to make conclusive statements about whether the toxicity of the mixture was higher or lower than expected or the cause of toxicity, because the single pesticides and different mixture combinations (of two, three, and four residues) were not tested. Thus, we based our comparison on ∑RQ. In Portugal the ∑RQ was 2.26 at the PEC exposure; however, the reproduction was almost completely inhibited at this exposure concentration, with a decrease of 93% compared with the solvent control (Figure 3). In France, the ∑RQ was 3.5 and the inhibition was 95%. In Spain/Italy, the RQ was 3.8 and the inhibition was 87%. The decrease in the reproduction rate above 90% seems exaggerated when considering that RQ was calculated using the NOEC. For example, by assessing approximately 200 toxicity data sets, Moore and Caux (1997) observed that most NOECs correspond to reductions up to 30% from control treatments.

When looking at the mixture composition, the two most toxic CSSs, Portugal and France, had in common the insecticide chlorantraniliprole and the fungicides boscalid and difenoconazole. Chlorantraniliprole binds to receptors in the muscles, affecting calcium release and causing paralysis of the muscle (Bentley et al., 2010). In the literature, the EC50 of chlorantraniliprole for effects on *F. candida* reproduction varied from 0.14–0.91 mg a.s./kg_dry weight_ soil (Ferreira et al., 2022; Lavtižar et al., 2016). These studies analyzed the influence of temperature and soil properties on chlorantraniliprole toxicity and therefore a wide range of EC50s was reported in both studies. Ferreira et al. (2022) observed a significant reduction of reproduction ≥ 1.2 mg a.s./kg_dry weight_ soil, whereas Lavtižar et al. (2016) observed a complete depletion of reproduction ≥ 1 mg a.s./kg_dry weight_ soil. Based on these results, the PEC exposure in our study seems to have caused higher effects than expected for a chlorantraniliprole concentration of 0.4 mg a.s./kg_dry weight_ soil. The fungicides boscalid and difenoconazole are not expected to cause any toxicity in springtails, with NOEC values > 250 mg a.s./kg_dry weight_ soil. Nevertheless, azole fungicides are known to inhibit the metabolization of insecticides, causing synergetic effects in mixture studies (Cedergreen, 2014). Triazole fungicides, including difenoconazole, are strong inhibitors of cytochrome P450 monooxygenases involved with the detoxification of chlorantraniliprole in honey bees, possibly causing synergetic effects (Haas et al., 2022). In this same study, the authors observed other fungicides, such as boscalid, caused only a weak inhibition of these enzymes, which would probably translate to negligible effects on in vivo models (Haas et al., 2022).

Studies on the effects of complex or whole mixtures of pesticides on springtails are scarce. In an interesting study by Panico et al. (2022), agricultural soils from conventional farms in France decreased the reproduction of *F. candida*. Moreover, the authors observed that the toxicity could not be solely explained by the single effects of the pesticides detected in the soil. The most toxic pesticide in the soil to *F. candida* was the insecticide imidacloprid; however, its concentration in the soil was five times lower than the NOEC reported in the literature by de Lima e Silva (2021). The authors attributed their results to a possible mixture effect.

Usually, the component-based approach and the whole mixture approach are used in mixture toxicity studies. In the component-based approach, the contribution of each component to the toxicity can be identified; however, it may be impractical when testing mixtures with several components. On the other hand, the whole mixture approach is an appropriate choice for complex mixtures; however, information is lost if the mixture composition changes with time. In our study, we followed a step-wise approach: (1) soils from 11 CSS were analyzed for approximately 200 pesticide residues (Knuth et al., 2024); (2) mixtures of up to five pesticides were selected based on their risk to soil invertebrates and frequency of detection in soil samples (Jegede et al., 2024); and finally, (3) the toxicity tests were conducted with the pesticide mixtures selected. In this way, we could study real and relatively complex mixtures of pesticides that are expected to cause higher risk to soil organisms at three different concentrations while keeping the number of test units to a minimum. Moreover, this approach links the exposure data obtained from the monitoring programs with hazard data obtained in the ecotoxicological tests.

A sustainable pesticide use in agriculture is key to guaranteeing the preservation of soil biodiversity (Beaumelle et al., 2023). Although no effects were observed in the springtail exposed to MEC treatments, suggesting that median pesticide levels in soils are likely to be safe for springtails, these results must be carefully interpreted. Real soil concentrations can often surpass the MEC and PEC. According to Knuth et al. (2024), 35% of the soil measured concentrations in the SPRINT monitoring campaign exceeded the PEC values, with some samples showing concentrations up to 10 times higher than PEC. Moreover, our study showed that pesticide mixtures can cause effects at environmentally relevant concentrations (i.e., the worst-case scenario PEC), which may be alarming.

## Conclusion

This work highlights the importance of assessing the effects of complex mixtures of pesticides. The toxicity in two CSSs (Portugal and France) seemed to be higher than expected assuming concentration additivity using the NOEC values of single pesticides. Although no effects were observed at MEC exposure, effects were observed at PEC exposure in several CSSs, which is worrying because these represent realistic exposure scenarios. More studies are needed to better understand whether the risk assessment of single pesticides is enough to protect soil species from exposure to multiple residues.

## Supplementary Material

vgaf057_Supplementary_Data

## Data Availability

The data that support the findings of this study are available from the corresponding author on reasonable request.
