# Supplementary material for: Effects of realistic pesticide mixtures on the springtail Folsomia candida
Source: Environ Toxicol Chem. 2025 Mar 3;44(5):1347–56. doi: 10.1093/etojnl/vgaf057 (PMC12047021; doi:10.1093/etojnl/vgaf057)
Supplement: vgaf057_Supplementary_Data [file vgaf057_supplementary_data.docx]

Supporting information

Effects of realistic pesticide mixtures on the springtail *Folsomia candida*

Paula S. Tourinho^1^, Zuzana Hochmanová^1^, Petr Kukučka^1^, Olukayode Jegede^2^, Vera Silva^2^, Virginia Aparicio^3^, Jakub Hofman^1^

^1^ RECETOX, Faculty of Science, Masaryk University, Kotlarska 2, Brno, Czech Republic

^2^ Soil Physics and Land Management Group, Wageningen University & Research, Droevendaalsesteeg 4, 6708, PB Wageningen, The Netherlands

^3^ INTA, Instituto Nacional de Tecnologías Agropecuaria. Ruta 226 km 73.5 Balcarce, Buenos Aires, Argentina

Table S1: Farming systems of the 11 case study sites.

| Study site | Crop | Farming system |
| --- | --- | --- |
| Spain (SP) | Vegetables | C, O |
| Portugal (PT) | Vineyards | C, O |
| France (FR) | Vineyards | C, O |
| Switzerland (CH) | Orchards | C, O |
| Italy (IT) | Vegetables | C, O |
| Croatia (HR) | Olives | C, O |
| Slovenia (SL) | Maize | C, O |
| Czechia (CZ) | Oilseed crops | C, O |
| The Netherlands (NL) | Root crops | C, O |
| Denmark (DK) | Cereals | C, O |
| Argentina (AR) | Cereals | C, I |

C=conventional, I=integrated, O=organic.

Table S2: Data of the pesticides selected for the ecotoxicological tests. DT50_typical_ were collected from the Pesticide Properties Database (<https://sitem.herts.ac.uk/aeru/ppdb/en/index.htm)>. Worst-scenario predicted environmental concentration (PEC) in soil and ecotoxicology outcomes values and their sources are shown. IN – insecticide, FU – fungicide, HB – herbicide, met-metabolite. No observed effect on reproduction (NOEC_rep_), 50% effect concentration on reproduction (EC_50 repr_), and 50% lethal concentration (LC_50_).

| Pesticide | Class |  | PEC | | Ecotoxicology | | | |
| --- | --- | --- | --- | --- | --- | --- | --- | --- |
|  |  | DT50 (days) | Value (mg/kg) | Source | Organism | Effect | Value (mg/kg) | Source |
| Acetamiprid | IN | 1.6 | 0.04 | EFSA, 2016a | *Folsomia candida* | NOEC_rep_ | 0.27 | EFSA, 2016a |
| AMPA | met | 114 | 2.03 | EFSA, 2015 | *Eisenia fetida* | NOEC_rep_ | 28.12 | PPDB, 2023 |
| Azoxystrobin | FU | 78 | 0.39 | EFSA, 2010a | *Eisenia fetida* | NOEC_rep_ | 20 | EFSA, 2010a |
| Bixafen | IN | 500 | 0.47 | EFSA, 2012 | *Hypoaspis aculeifer* | NOEC_rep_ | 6.15 | EFSA, 2012 |
| Boscalid | FU | 484 | 0.78 | EC, 2008 | *Eisenia fetida* | NOEC_rep_ | 1.20 | EC, 2008 |
| Chlorantraniliprole | IN | 597 | 0.43 | EFSA, 2013a | *Folsomia candida* | NOEC_rep_ | 0.39 | EFSA, 2013a |
| Cyflufenamid | FU | 34 | 0.02 | EFSA, 2009a | *Folsomia candida* | NOEC_rep_ | 0.0178 | EFSA, 2009a |
| Deltamethrin | IN | 58 | 0.02 | EC, 2018 | *Eisenia fetida* | NOEC_rep_ | 0.165 | PPDB, 2023 |
| Difenoconazole | FU | 133 | 0.22 | EFSA, 2011a | *Eisenia fetida* | NOEC_rep_ | 0.2 | EFSA, 2011a |
| Diflufenican | HB | 94 | 0.24 | EFSA, 2007 | *Hypoaspis aculeifer* | EC_50 repr_ | 5.4 | EFSA, 2007a |
| Dimoxystrobin | FU | 232 | 0.04 | EFSA, 2022 | *Eisenia fetida* | NOEC_rep_ | 0.025 | EFSA, 2022 |
| Fluopyram | FU | 309 | 0.43 | EFSA, 2013b | *Eisenia fetida* | NOEC_rep_ | 11.42 | EFSA, 2013b |
| Glyphosate | HB | 17 | 5.76 | EFSA, 2015 | *Eisenia fetida* | NOEC_rep_ | > 21.31 | EFSA, 2015 |
| Lambda-cyhalothrin | IN | 174 | 0.04 | EFSA, 2014a | *Porcellionides pruinosus* | EC_50 repr_ | 0.13 | US-EPA, 2022 |
| Methoxyfenozide | IN | 456 | 0.22 | EFSA, 2017 | *Folsomia candida* | NOEC_rep_ | 0.6 | EFSA, 2017 |
| Myclobutanil | FU | 560 | 0.42 | EFSA, 2010b | *Eisenia fetida* | NOEC_rep_ | 10.3 | EFSA, 2010b |
| Oxyfluorfen | HB | 35 | 2.5 | EFSA, 2010c | *Folsomia candida* | NOEC_rep_ | 0.125 | EFSA, 2010c |
| Pendimethalin | HB | 182 | 2.13 | EFSA, 2016b | *Eisenia fetida* | NOEC_rep_ | 33.45 | EFSA, 2016b |
| Phosmet | IN | 3.2 | 0.41 | EFSA, 2020 | *Folsomia candida* | NOEC_rep_ | 0.81 | EFSA, 2020 |
| Pirimicarb | IN | 73 | 0.34 | EFSA, 2005 | *Eisenia fetida* | NOEC_rep_ | 5.46 | United Kingdom, 2017 |
| Prosulfocarb | HB | 12 | 5.33 | EFSA, 2007b | *Eisenia fetida* | LC_50_ | 71.8 | EFSA, 2007b |
| S-metolachlor | HB | 90 | 1.92 | Germany, 2018 | *Eisenia fetida* | NOEC_50_ | 53.3 | Germany, 2018 |
| Tebuconazole | FU | 63 | 0.18 | EFSA, 2014b | *Eisenia fetida* | NOEC_rep_ | 10 | EFSA, 2014b |
| Terbuthylazine | HB | 72 | 1.12 | EFSA, 2011b | *Eisenia fetida* | LC_50_ | >141.7 | EFSA, 2011b |
| Thiophanate-methyl | FU | 0.5 | 5.53 | EFSA, 2016c | *Eisenia fetida* | NOEC_rep_ | 1.6 | EFSA, 2016c |

Table S3: No observed effect concentration (NOEC) for effects on springtail reproduction of pesticides as active substance. For pesticides with LogPow>2, the NOEC values corrected by EFSA are indicated as NOEC_corr_, and the NOEC values where a factor of 2 was applied are indicated as NOEC_fact_. * indicates NOEC values for formulations.

| CSS | Pesticides | NOEC | mg/kg | Source |
| --- | --- | --- | --- | --- |
| SP/IT | Oxyfluorfen | NOEC_corr_ | 1.25 | EFSA, 2010c |
|  | Chlorantraniliprole | NOEC_fact_ | 0.20 | EFSA, 2013a |
|  | Difenoconazole | NOEC_fact_ | 250 | EFSA, 2011a |
|  | Lambda-Cyhalothrin | NOEC_corr_ | 2.73 | EFSA, 2014a |
|  | Boscalid | NOEC_fact_ | 500 | Slovakia, 2018 |
| PT | Chlorantraniliprole | NOEC_fact_ | 0.20 | EFSA, 2013a |
|  | Boscalid | NOEC_fact_ | 500 | Slovakia, 2018 |
|  | Glyphosate | NOEC | 587 | EFSA, 2015 |
|  | Difenoconazole | NOEC_fact_ | 250 | EFSA, 2011 |
|  | AMPA | NOEC | 315 | EFSA, 2015 |
| FR | Chlorantraniliprole | NOEC_fact_ | 0.20 | EFSA, 2013a |
|  | Boscalid | NOEC_fact_ | 500 | Slovakia, 2018 |
|  | Difenoconazole | NOEC_fact_ | 250 | EFSA, 2011 |
|  | Cyflufenamid* | NOEC_corr_ | 0.02 | United Kingdom, 2006 |
|  | Glyphosate | NOEC | 587 | EFSA, 2015 |
| SW | Difenoconazole | NOEC_fact_ | 250 | EFSA, 2011 |
|  | Methoxyfenozide | NOEC_corr_ | 0.64 | EFSA, 2017 |
|  | Myclobutanil* | NOEC_corr_ | 10.2 | EFSA, 2010b |
|  | AMPA | NOEC | 315 | EFSA, 2015 |
|  | Pirimicarb* | NOEC | 16 | United Kingdom, 2017 |
| HR | Boscalid | NOEC_fact_ | 500 | Slovakia, 2018 |
|  | Phosmet | NOEC_corr_ | 0.81 | EFSA, 2020 |
|  | Acetamiprid | NOEC | 0.27 | EFSA, 2016a |
|  | AMPA | NOEC | 315 | EFSA, 2015 |
|  | Deltamethrin | NOEC_corr_ | 16 | United Kingdom, 2018 |
| SL | Bixafen* | NOEC_corr_ | 7.74 | EFSA, 2012 |
|  | S-Metolachlor | NOEC_fact_ | 130 | Germany, 2018 |
|  | AMPA | NOEC | 315 | EFSA, 2015 |
|  | Tebuconazole | NOEC_corr_ | 250 | EFSA, 2014b |
|  | Terbuthylazine* | NOEC_corr_ | 21.12 | United Kingdom, 2007 |
| CZ | Boscalid | NOEC_fact_ | 500 | Slovakia, 2018 |
|  | Thiophanate-methyl* | NOEC | 100 | EFSA, 2016c |
|  | Lambda-Cyhalothrin | NOEC_corr_ | 2.73 | EFSA, 2014a |
|  | Dimoxystrobin | NOEC_corr_ | 500 | EFSA, 2022 |
|  | Azoxystrobin | NOEC_corr_ | 25 | EFSA, 2009b |
| NT | Lambda-Cyhalothrin | NOEC_corr_ | 2.73 | EFSA, 2014a |
|  | Boscalid | NOEC_fact_ | 500 | Slovakia, 2018 |
|  | Azoxystrobin | NOEC_corr_ | 25 | EFSA, 2009 |
|  | Bixafen* | NOEC_corr_ | 7.74 | EFSA, 2012 |
|  | Prosulfocarb | - | - | - |
| *Cont.* |  |  |  |  |
| CSS | Pesticides | NOEC | mg/kg | Source |
| DE | Boscalid | NOEC_fact_ | 500 | Slovakia, 2018 |
|  | AMPA | NOEC | 315 | EFSA, 2015 |
|  | Diflufenican* | NOEC_corr_ | 219 | EFSA, 2007 |
|  | Fluopyram* | NOEC_corr_ | 104 | EFSA, 2013b |
|  | Pendimethalin | NOEC_corr_ | 193 | EFSA, 2016b |
| AR | Glyphosate | NOEC | 587 | EFSA, 2015 |
|  | Lambda-Cyhalothrin | NOEC_corr_ | 2.73 | EFSA, 2014a |
|  | AMPA | NOEC | 315 | EFSA, 2015 |
|  | Azoxystrobin | NOEC_corr_ | 25 | EFSA, 2009b |
|  | Methoxyfenozide | NOEC_corr_ | 0.64 | EFSA, 2017 |

Table S4: Risk quotient (RQ) calculated as exposure concentration divided by NOEC for effects on springtail reproduction of pesticides.

| CSS | Pesticides | RQ | | |
| --- | --- | --- | --- | --- |
|  |  | MEC | PEC | 5xPEC |
| SP/IT | Oxyfluorfen | 0.11/0.04 | 1.54 | 7.68 |
|  | Chlorantraniliprole | 0.17/0.07 | 2.25 | 11.23 |
|  | Difenoconazole | 0.00/0.00 | 0.00 | 0.00 |
|  | lambda Cyhalothrin | 0.01/0.00 | 0.01 | 0.06 |
|  | Boscalid | 0.00/0.00 | 0.00 | 0.00 |
| PT | Chlorantraniliprole | 0.03 | 2.25 | 11.23 |
|  | Boscalid | 0.00 | 0.00 | 0.00 |
|  | Glyphosate | 0.00 | 0.01 | 0.05 |
|  | Difenoconazole | 0.00 | 0.00 | 0.00 |
|  | AMPA | 0.01 | 0.01 | 0.03 |
| FR | Chlorantraniliprole | 0.07 | 2.25 | 11.23 |
|  | Boscalid | 0.00 | 0.00 | 0.00 |
|  | Difenoconazole | 0.00 | 0.00 | 0.00 |
|  | Cyflufenamid | 0.23 | 1.32 | 6.60 |
|  | Glyphosate | 0.00 | 0.01 | 0.05 |
| SW | Difenoconazole | 0.00 | 0.00 | 0.00 |
|  | Methoxyfenozide | 0.01 | 0.14 | 0.71 |
|  | Myclobutanil | 0.00 | 0.07 | 0.33 |
|  | AMPA | 0.00 | 0.01 | 0.03 |
|  | Pirimicarb | 0.00 | 0.01 | 0.05 |
| HR | Boscalid | 0.00 | 0.00 | 0.00 |
|  | Phosmet | 0.01 | 0.49 | 2.47 |
|  | Acetamiprid | 0.04 | 1.07 | 5.37 |
|  | AMPA | 0.00 | 0.01 | 0.03 |
|  | deltamethrin | 0.00 | 0.00 | 0.01 |
| SL | Bixafen | 0.00 | 0.02 | 0.11 |
|  | Metolachlor (S) | 0.00 | 0.01 | 0.07 |
|  | AMPA | 0.00 | 0.01 | 0.03 |
|  | Tebuconazole | 0.00 | 0.00 | 0.00 |
|  | Terbuthylazine | 0.00 | 0.05 | 0.27 |
| CZ | Boscalid | 0.00 | 0.00 | 0.00 |
|  | Thiophanate-methyl | 0.00 | 0.06 | 0.28 |
|  | lambda Cyhalothrin | 0.00 | 0.01 | 0.06 |
|  | Dimoxystrobin | 0.00 | 0.00 | 0.00 |
|  | Azoxystrobin | 0.00 | 0.02 | 0.08 |
| NT | lambda Cyhalothrin | 0.00 | 0.01 | 0.06 |
|  | Boscalid | 0.00 | 0.00 | 0.00 |
|  | Azoxystrobin | 0.00 | 0.02 | 0.08 |
|  | Bixafen | 0.00 | 0.02 | 0.11 |
|  | Prosulfocarb | - | - | - |

|  |  |  |  |  |
| --- | --- | --- | --- | --- |
| *Cont.* |  |  |  |  |
| CSS | Pesticides | RQ | | |
|  |  | MEC | PEC | 5xPEC |
| DE | Boscalid | 0.00 | 0.00 | 0.00 |
|  | AMPA | 0.00 | 0.01 | 0.03 |
|  | Diflufenican | 0.00 | 0.00 | 0.00 |
|  | Fluopyram | 0.00 | 0.00 | 0.01 |
|  | Pendimethalin | 0.00 | 0.01 | 0.06 |
| AR | Glyphosate | 0.00 | 0.01 | 0.05 |
|  | lambda-Cyhalothrin | 0.00 | 0.01 | 0.06 |
|  | AMPA | 0.01 | 0.01 | 0.03 |
|  | Azoxystrobin | 0.00 | 0.02 | 0.08 |
|  | Methoxyfenozide | 0.02 | 0.14 | 0.71 |

Table S5: Measured concentration of pesticides in soil samples 11 case study sites (CSS). Samples from control (CT), solvent control (CTs), median measured environmental concentration (MEC), predicted environmental concentration (PEC), and 5 times PEC (5xPEC) were measured after spiking (Initial) and after 28 days (Final) of the reproduction tests. The initial concentration are also expressed as % of the nominal concentrations.

| CSS | Pesticide | CT | | CTs | | MEC | | | PEC | | | 5xPEC | | |
| --- | --- | --- | --- | --- | --- | --- | --- | --- | --- | --- | --- | --- | --- | --- |
|  |  | Initial | Final | Initial | Final | Initial | % nominal | Final | Initial | (% nominal) | Final | Initial | (% nominal) | Final |
| SP^a^ | Oxyfluorfen | <LOQ | <LOQ | <LOQ | <LOQ | 0.003^a^  0.04^b^ | 2.51^a^  77.0^b^ | 0.003^a^  0.04^b^ | 0.48 | 25.2 | 0.40 | 7.79 | 81.1 | 7.49 |
| IT^b^ | Chlorantraniliprole | <LOQ | <LOQ | <LOQ | <LOQ | 0.012^a^  0.001^b^ | 36.5^a^  66.0^b^ | 0.01^a^  0.01^b^ | 0.36 | 82.9 | 0.31 | 3.58 | 163.4 | 3.43 |
|  | Difenoconazole | <LOQ | <LOQ | <LOQ | <LOQ | 0.0002^a^ 0.01^b^ | 0.78^a^  70.4^b^ | 0.003^a^  0.01^b^ | 0.05 | 33.4 | 0.04 | 0.52 | 77.3 | 0.48 |
|  | λ-Cyhalothrin | <LOQ | <LOQ | <LOQ | <LOQ | 0.007^a^  0.003^b^ | 26.9^a^  53.3^b^ | 0.0005^a^  0.002^b^ | 0.00 | 14.6 | 0.001 | 0.03 | 20.7 | 0.00 |
|  | Boscalid | <LOQ | <LOQ | <LOQ | <LOQ | 0.004^a^ 0.01^b^ | 17.2^a^  35.3^b^ | 0.003^a^  0.01^b^ | 0.16 | 39.6 | 0.14 | 1.33 | 67.0 | 1.26 |
| PT | Chlorantraniliprole | <LOQ | <LOQ | <LOQ | <LOQ | 0.00 | 64.7 | 0.00 | 0.34 | 77.1 | 0.35 | 1.76 | 80.5 | 1.89 |
|  | Boscalid | 0.01 | 0.00 | 0.00 | 0.00 | 0.15 | 83.4 | 0.16 | 0.35 | 87.7 | 0.35 | 1.50 | 76.0 | 1.57 |
|  | Glyphosate | 0.08 | <LOQ | 0.07 | 0.12 | 0.62 | 78.7 | 0.58 | 4.96 | 86.1 | 4.04 | 25.55 | 88.7 | 9.43 |
|  | Difenoconazole | 0.00 | 0.00 | 0.00 | 0.00 | 0.01 | 120.7 | 0.01 | 0.09 | 66.7 | 0.08 | 0.53 | 78.5 | 0.53 |
|  | AMPA | 0.19 | 0.12 | 0.18 | 0.19 | 1.28 | 66.9 | 1.16 | 2.60 | 127.9 | 2.90 | 10.85 | 106.6 | 12.50 |
| FR | Chlorantraniliprole | <LOQ | <LOQ | <LOQ | <LOQ | 0.01 | 0.01 | 75.3 | 0.33 | 75.9 | 0.34 | 2.60 | 118.8 | 2.46 |
|  | Boscalid | 0.01 | 0.004 | 0.005 | 0.003 | 0.03 | 0.03 | 119.1 | 0.35 | 87.2 | 0.35 | 2.26 | 114.0 | 2.06 |
|  | Difenoconazole | 0.004 | 0.003 | 0.005 | 0.003 | 0.01 | 0.01 | 105.6 | 0.09 | 65.1 | 0.08 | 0.67 | 99.6 | 0.56 |
|  | Cyflufenamid | <LOQ | <LOQ | <LOQ | <LOQ | 0.00 | 0.00 | 82.0 | 0.02 | 69.5 | 0.01 | 0.09 | 77.6 | 0.04 |
|  | Glyphosate | 0.08 | <LOQ | 0.07 | 0.12 | 0.26 | 0.36 | 138.6 | 4.76 | 82.7 | 3.66 | 27.46 | 95.4 | 9.63 |
| SW | Difenoconazole | <LOQ | <LOQ | 0.002 | 0.002 | 0.01 | 0.00 | 31.7 | 0.04 | 26.5 | 0.04 | 0.51 | 75.6 | 0.47 |
|  | Methoxyfenozide | <LOQ | <LOQ | <LOQ | <LOQ | 0.01 | 0.00 | 28.2 | 0.00 | 2.9 | 0.00 | 0.36 | 79.9 | 0.34 |
|  | Myclobutanil | <LOQ | <LOQ | <LOQ | <LOQ | 0.01 | 0.00 | 29.6 | 0.30 | 44.8 | 0.29 | 2.57 | 76.5 | 2.38 |
|  | AMPA | 0.15 | 0.14 | 0.13 | 0.14 | 0.34 | 0.34 | 99.4 | 1.47 | 72.4 | 1.38 | 6.64 | 65.2 | 6.48 |
|  | Pirimicarb | <LOQ | <LOQ | <LOQ | <LOQ | 0.02 | 0.01 | 25.7 | 0.04 | 25.7 | 0.03 | 0.49 | 61.7 | 0.38 |
| HR | Boscalid | <LOQ | <LOQ | <LOQ | <LOQ | 0.13 | 0.13 | 97.3 | 0.54 | 135.7 | 0.53 | 1.81 | 91.5 | 2.08 |
|  | Phosmet | <LOQ | <LOQ | <LOQ | <LOQ | 0.01 | <LOQ | - | 0.37 | 92.4 | 0.03 | 2.01 | 100.7 | 0.13 |
|  | Acetamiprid | <LOQ | <LOQ | <LOQ | <LOQ | 0.01 | 0.01 | 55.7 | 0.22 | 74.7 | 0.01 | 1.25 | 85.9 | 0.02 |
|  | AMPA | 0.14 | 0.10 | 0.13 | 0.07 | 0.52 | 0.58 | 111.6 | 1.65 | 81.2 | 1.35 | 8.21 | 80.6 | 7.65 |
|  | Deltamethrin | <LOQ | <LOQ | <LOQ | <LOQ | 0.04 | 0.057 | 159.7 | 0.052 | 234.4 | 0.030 | 0.319 | 289.6 | <LOQ |
| SL | Bixafen | <LOQ | <LOQ | <LOQ | <LOQ | 0.01 | 0.01 | 124.6 | 0.11 | 67.8 | 0.12 | 0.74 | 88.6 | 0.67 |
|  | Metolachlor (S) | <LOQ | <LOQ | <LOQ | <LOQ | 0.04 | 0.02 | 42.2 | 1.07 | 55.5 | 1.04 | 8.40 | 87.5 | 7.60 |
|  | AMPA | 0.14 | 0.10 | 0.13 | 0.07 | 0.07 | 0.24 | 345.8 | 1.99 | 97.6 | 1.52 | 8.15 | 80.0 | 7.11 |
|  | Tebuconazole | <LOQ | <LOQ | <LOQ | <LOQ | 0.01 | <LOQ | - | 0.12 | 64.8 | 0.13 | 0.70 | 75.7 | 0.73 |
|  | Terbuthylazine | <LOQ | <LOQ | <LOQ | <LOQ | 0.01 | 0.00 | 52.2 | 0.64 | 56.9 | 0.85 | 5.28 | 93.9 | 5.21 |
| CZ | Boscalid | <LOQ | <LOQ | <LOQ | <LOQ | 0.02 | 0.01 | 47.7 | 0.01 | 1.8 | 0.14 | 1.29 | 65.1 | 1.81 |
|  | Thiophanate-methyl | <LOQ | <LOQ | <LOQ | <LOQ | 0.16 | 0.03 | 17.1 | 3.34 | 60.4 | 0.30 | 42.90 | 155.1 | 1.69 |
|  | λ-Cyhalothrin | <LOQ | <LOQ | <LOQ | <LOQ | 0.00 | 0.01 | 433.3 | 0.11 | 331.0 | 0.01 | 0.53 | 326.9 | 0.04 |
|  | Dimoxystrobin | <LOQ | <LOQ | <LOQ | <LOQ | 0.02 | 0.01 | 38.4 | 0.02 | 32.4 | 0.01 | 0.07 | 29.5 | 0.14 |
|  | Azoxystrobin | <LOQ | <LOQ | <LOQ | <LOQ | 0.01 | 0.00 | 30.5 | 0.12 | 30.6 | 0.10 | 1.15 | 58.4 | 1.02 |
| NT | λ-Cyhalothrin | <LOQ | <LOQ | <LOQ | <LOQ | 0.00 | <LOQ | - | 0.04 | 111.7 | 0.01 | 0.32 | 197.6 | 0.07 |
|  | Boscalid | <LOQ | <LOQ | <LOQ | <LOQ | 0.02 | 0.02 | 88.2 | 0.13 | 32.9 | 0.16 | 1.29 | 65.0 | 1.31 |
|  | Azoxystrobin | <LOQ | <LOQ | <LOQ | <LOQ | 0.02 | 0.01 | 48.4 | 0.10 | 25.1 | 0.10 | 1.07 | 54.3 | 0.97 |
|  | Bixafen | <LOQ | <LOQ | <LOQ | <LOQ | 0.02 | 0.01 | 26.0 | 0.04 | 23.1 | 0.05 | 0.63 | 75.5 | 0.65 |
|  | Prosulfocarb | <LOQ | <LOQ | <LOQ | <LOQ | 0.06 | 0.01 | 19.0 | 1.73 | 32.4 | 0.44 | 23.34 | 87.5 | 1.87 |
| DE | Boscalid | <LOQ | <LOQ | <LOQ | <LOQ | 0.01 | 0.01 | 75.8 | 0.40 | 100.4 | - | 1.78 | 89.8 | 1.70 |
|  | AMPA | 0.14 | 0.10 | 0.13 | 0.07 | 0.12 | 0.33 | 285.6 | 1.94 | 95.4 | 1.35 | 7.38 | 72.5 | 7.65 |
|  | Diflufenican | <LOQ | <LOQ | <LOQ | <LOQ | 0.02 | 0.02 | 136.3 | 0.17 | 66.1 | 0.17 | 0.83 | 66.2 | 0.82 |
|  | Fluopyram | <LOQ | <LOQ | <LOQ | <LOQ | 0.01 | 0.00 | 52.1 | 0.19 | 72.5 | 0.19 | 1.27 | 97.8 | 1.49 |
|  | Pendimethalin | <LOQ | <LOQ | <LOQ | <LOQ | 0.05 | 0.03 | 55.6 | 1.44 | 67.6 | - | 7.60 | 71.2 | - |
| AR | Glyphosate | 0.05 | - | 0.07 | - | 0.54 | 0.62 | 113.5 | 6.44 | 111.8 | - | 30.58 | 106.2 | - |
|  | λ-Cyhalothrin | <LOQ | - | <LOQ | - | 0.01 | 0.01 | 92.8 | 0.05 | 143.6 | - | 0.28 | 173.8 | - |
|  | AMPA | 0.09 | - | 0.12 | - | 1.33 | 1.05 | 79.0 | 1.75 | 86.0 | - | 9.03 | 88.7 | - |
|  | Azoxystrobin | <LOQ | - | <LOQ | - | 0.01 | 0.01 | 58.0 | 0.27 | 68.5 | - | 1.67 | 84.5 | - |
|  | Methoxyfenozide | <LOQ | - | <LOQ | - | 0.01 | 0.01 | 56.0 | 0.06 | 69.1 | - | 0.31 | 68.6 | - |


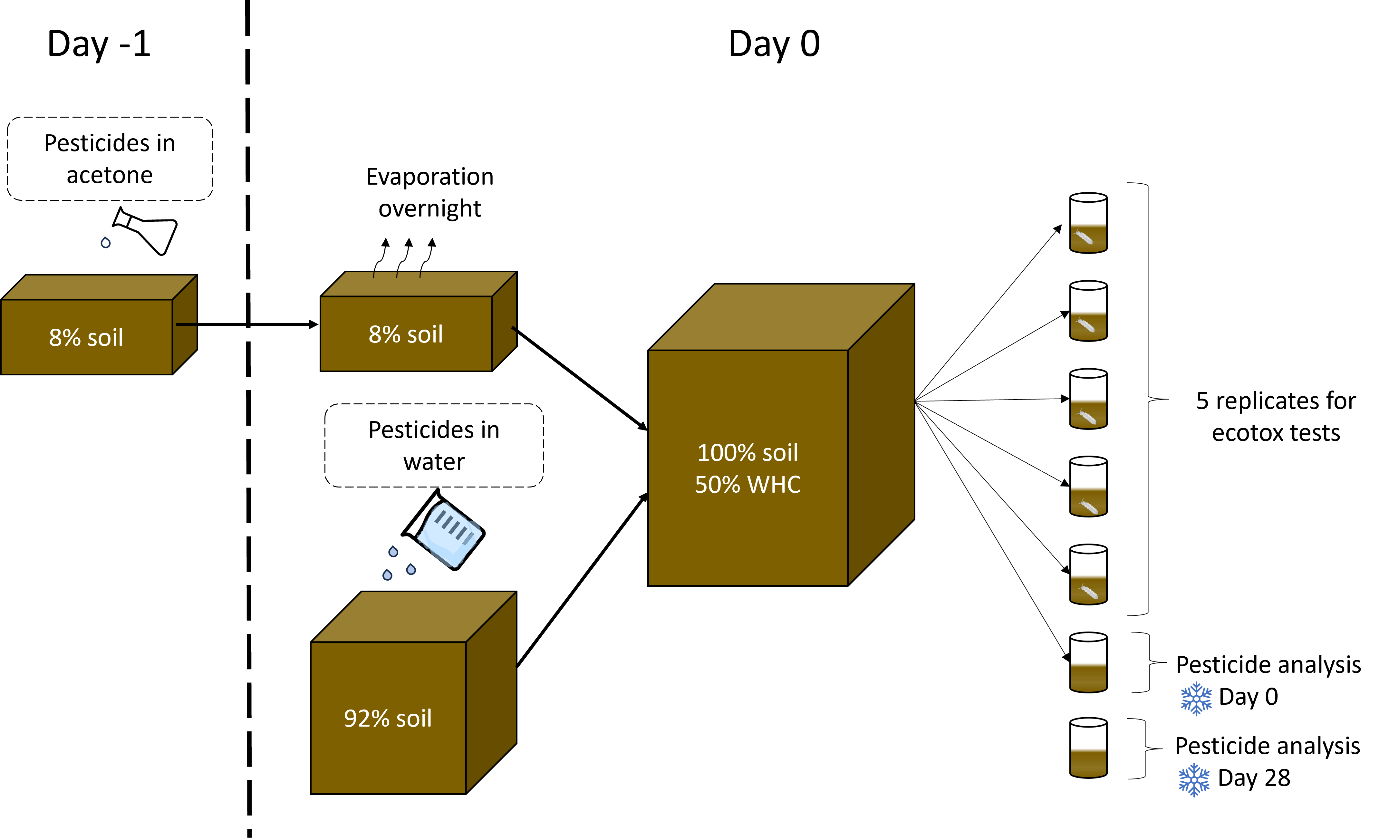


Figure S1: Spiking procedure of soil with current-use pesticides (CUP). CUP soluble in acetone were added to 8% soil one day before the experiment starts and left to evaporate overnight. CUP soluble in water were added to the remaining 92% soil at the same time as adjust WHC to 50% of the total soil amount. The two batches mixed and divided in 5 replicates for the tests and two extra replicates for the pesticide analysis (initial: immediately stored at -20 °C after spiking; final: incubated for 28 days).


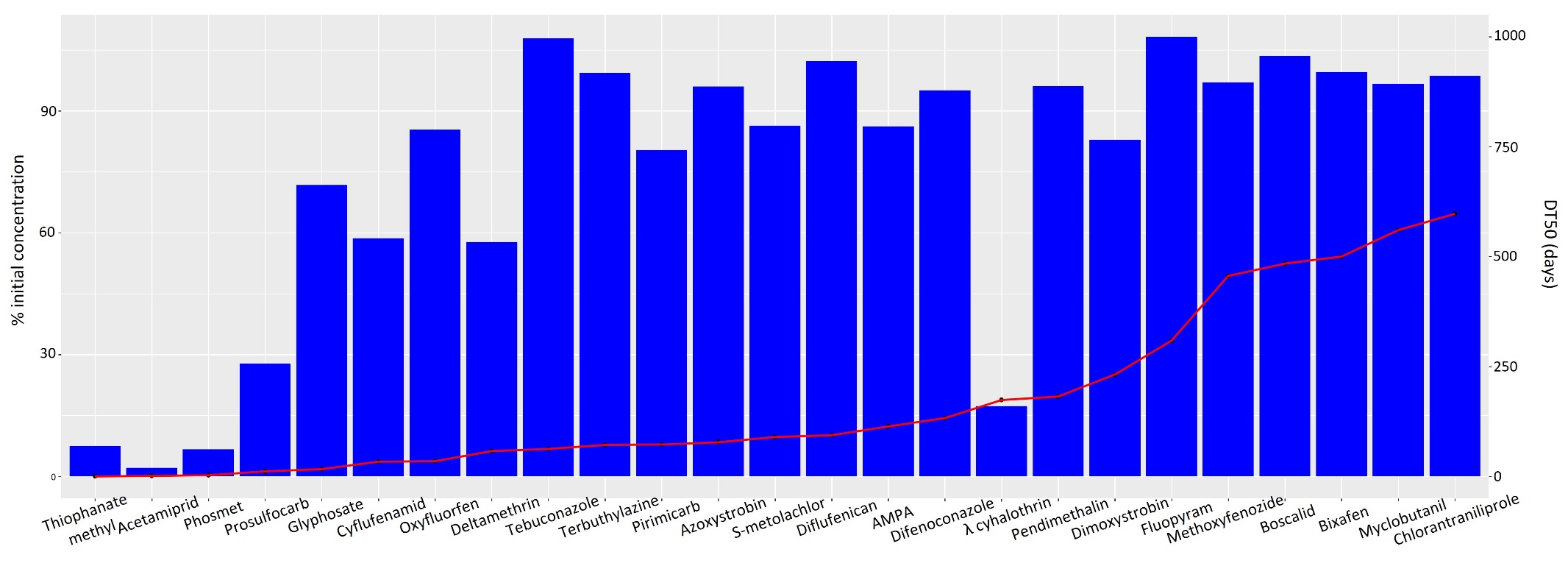


Figure S2: Mean final concentration of pesticides measured after 28 days, expressed as % of initial measured concentration. The DT50 (typical) of the pesticides according to PPDB database.


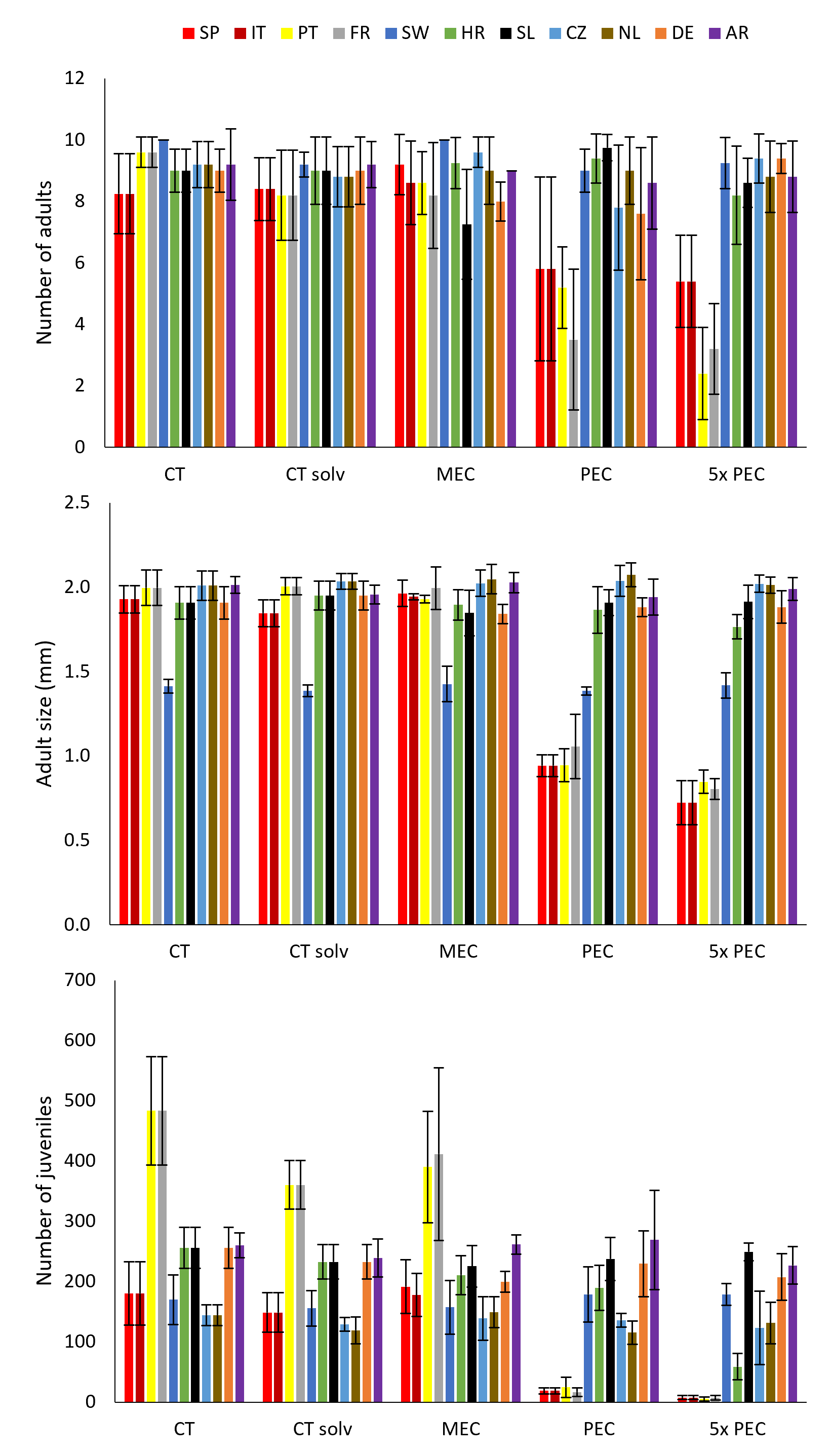


Figure S2: Number of adult (top), size of surviving adults (middle), and number of juveniles (bottom) of springtails exposed to negative control (CT), solvent control (CT solv), median environmental concentration (MEC), predicted environmental concentration (PEC) and 5 times PEC (5xPEC) after 4 weeks of exposure.

References

EC (European Commission), 2008. Review report for the active substance boscalid. Finalised in the Standing Committee on the Food Chain and Animal Health at its meeting on 22 January 2008 in view of the inclusion of boscalid in Annex I of Council Directive 91/414/EEC. SANCO/3919/2007-rev.5, 21 January 2008.

EC (European Commission), 2018. Draft Renewal Assessment Report prepared according to the Commission Regulation (EU) No. 1107/2009. Deltamethrin. Volume 3 – B.8.

EFSA (European Food Safety Authority), 2005. Conclusion regarding the peer review of the pesticide risk assessment of the active substance Pirimicarb. EFSA Journal **3**, 43.

EFSA (European Food Safety Authority), 2007a. Conclusion on the peer review of the pesticide risk assessment of the active substance Diflufenican. *EFSA Scientific Report* (2007) 122, 1-84 pp.

EFSA (European Food Safety Authority), 2007b. Conclusion regarding the peer review of the pesticide risk assessment of the active substance prosulfocarb EFSA Scientific Report (2007) 111, 1-81 pp.

EFSA (European Food Safety Authority), 2009a. Conclusion on pesticide peer review regarding the risk assessment of the active substance cyflufenamid. *EFSA Scientific Report* (2009) 258, 1-99 pp.

EFSA (European Food Safety Authority), 2009b. Council directive 91/414/EEC. Azoxystrobin. Report and proposed decision of the United Kingdom made to the European Commission under commission regulation 737/2007. Accessed via http://dar.efsa.europa.eu/dar-web/provision, 2009.

EFSA (European Food Safety Authority), 2010a. European Food Safety Authority; Conclusion on the peer review of the pesticide risk assessment of the active substance azoxystrobin. EFSA Journal 2010; 8(4):15421542, 110 pp. doi:10.2903/j.efsa.2010.1542

EFSA (European Food Safety Authority), 2010b. Conclusion on the peer review of the pesticide risk assessment of the active substance myclobutanil. EFSA Journal 2010 8(10) 1682, 83pp. doi: 10.2903/jefsa.2010.1682

EFSA (European Food Safety Authority), 2010c. Conclusion on the peer review of the pesticide risk assessment of the active substance oxyfluorfen. EFSA Journal 2010;8(11):1906, 78 pp. doi:10.2903/j.efsa.2010.1906

EFSA (European Food Safety Authority), 2011a. European Food Safety Authority; Conclusion on the peer review of the pesticide risk assessment of the active substance difenoconazole. EFSA Journal 2011;9(1):1967, 71 pp. doi:10.2903/j.efsa.2011.1967

EFSA (European Food Safety Authority), 2011b. European Food Safety Authority; Conclusion on the peer review of the pesticide risk assessment of the active substance terbuthylazine. EFSA Journal 2011; 9(1):1969, 133 pp. doi:10.2903/j.efsa.2011.1969.

EFSA (European Food Safety Authority), 2012. Conclusion on the peer review of the pesticide risk assessment of the active substance bixafen. EFSA Journal 2012;10(11):2917, 87 pp. doi:10.2903/j.efsa.2012.2917

EFSA (European Food Safety Authority), 2013a. European Food Safety Authority; Conclusion on the peer review of the pesticide risk assessment of the active substance [chlorantraniliprole]. EFSA Journal 2013;11(6):3143, 107 pp. doi:10.2903/j.efsa.2013.3143

EFSA (European Food Safety Authority), 2013b. Conclusion on the peer review of the pesticide risk assessment of the active substance fluopyram. EFSA Journal 2013;11(4):3052, [76 pp. doi:10.2903/j.efsa.2013.3052.

EFSA (European Food Safety Authority), 2014a. Conclusion on the peer review of the pesticide risk assessment of the active substance lambda-cyhalothrin. EFSA Journal 2014;12(5):3677, 170 pp. doi:10.2903/j.efsa.2014.3677

EFSA (European Food Safety Authority), 2014b. Conclusion on the peer review of the pesticide risk assessment of the active substance tebuconazole. EFSA Journal 2014;12(1):3485, 98 pp. doi:10.2903/j.efsa.2014.3485

EFSA (European Food Safety Authority), 2015. Conclusion on the peer review of the pesticide risk assessment of the active substance glyphosate. EFSA Journal 2015;13(11):4302, 107 pp. doi:10.2903/j.efsa.2015.4302

EFSA (European Food Safety Authority), 2016a. Conclusion on the peer review of the pesticide riskssessment of the active substance acetamiprid. EFSA Journal 2016;14(11):4610, 91 pp. doi:10.2903/j.efsa.2016.4610

EFSA (European Food Safety Authority), 2016b. Conclusion on the peer review of the pesticide risk assessment of the active substance pendimethalin. EFSA Journal 2016;14(3):4420, 212 pp. doi:10.2903/j.efsa.2016.4420

EFSA (European Food Safety Authority), 2016c. Conclusion on the peer review of the pesticide risk assessment of the active substance thiophanate-methyl. EFSA Journal 2018;16(1):5133, 123 pp. doi:10.2903/j.efsa.2018.5133

EFSA (European Food Safety Authority), 2017. Conclusion on the peer review of the pesticide risk assessment of the active substance methoxyfenozide. EFSA Journal 2017;15(8):4978, 30 pp. doi:10.2903/j.efsa.2017.4978

EFSA (European Food Safety Authority), 2020. Conclusion on the peer review of the pesticide risk assessment of the active substance phosmet. EFSA Journal 2020;18(9):6237, 134 pp. doi:10.2903/j.efsa.2020.6237

EFSA (European Food Safety Authority) 2022. Statement concerning the assessment of environmental fate and behaviour and ecotoxicology in the context of the pesticides peer review of the active substance dimoxystrobin. EFSA Journal 2022;20(11):7634, 90 pp. https://doi.org/ 10.2903/j.efsa.2022.7634

Germany, 2018. Renewal Assessment Report (RAR) on the active substance S-metolachlor prepared by the rapporteur Member State Germany, in the framework of Commission Implementing Regulation (EU) No 844/2012, September 2018.

Pesticide Properties DataBase (PPDB), 2023. https://sitem.herts.ac.uk/aeru/ppdb/en/index.htmdatabase. Accessed: November, 2023.

Slovakia, 2018. Draft renewal assessment report on the active substance boscalid prepared by the rapporteur Member State Slovakia in the framework of Commission Regulation (EU) No 1107/2009, November 2018.

United Kingdom, 2006. Draft assessment report on the active substance cyflufenamid prepared by the rapporteur Member State the United Kingdom in in the framework of Council Directive 91/414/EEC, June, 2006.

United Kingdom, 2007. Draft assessment report on the active substance terbuthylazine prepared by the rapporteur Member State the United Kingdom in in the framework of Council Directive 91/414/EEC, November, 2007.

United Kingdom, 2017. Draft renewal assessment report on the active substance pirimicarb prepared by the rapporteur Member State United Kingdom in the framework of Commission Regulation (EU) No 1107/2009, December 2017.

United Kingdom, 2018. Draft renewal assessment report on the active substance deltamethrin prepared by the rapporteur Member State United Kingdom in the framework of Commission Regulation (EU) No 1107/2009, February 2018.

USEPA, 2022. Ecotox Database. <https://www.epa.gov/ecotox>. Access on June 2022.
